# Trauma Profile in Shahroud: An 8-Year Report of a Hospital-Based Trauma Registry

**DOI:** 10.34172/jrhs.2024.142

**Published:** 2024-03-15

**Authors:** Mahgol Sadat Hassan Zadeh Tabatabaei, Vali Baigi, Mohammadreza Zafarghandi, Vafa Rahimi-Movaghar, Salman Daliri, Sara Mirzamohamadi, Armin Khavandegar, Khatereh Naghdi, Payman Salamati

**Affiliations:** ^1^Sina Trauma and Surgery Research Center, Sina Hospital, Tehran University of Medical Sciences, Tehran, Iran; ^2^Clinical Research Development Unit, Imam Hossein Hospital, Shahroud University of Medical Sciences, Shahroud, Iran; ^3^Research Center for War-affected People, Tehran University of Medical Sciences, Tehran, Iran

**Keywords:** Wounds and Injuries, Registries, Accidents, Traffic

## Abstract

**Background:** Trauma is a significant public health concern in Iran, with high mortality and morbidity rates. This study aimed to assess trauma patients’ profiles in Shahroud, Iran.

**Study Design:** A cross-sectional study.

**Methods:** The study involved trauma patients who met specific criteria at Imam Hossein hospital in Shahroud, Iran, between 2016 and 2023, using the National Trauma Registry of Iran (NTRI). The relationship between injury characteristics and the cause of injury was analyzed using chi-square test and post hoc analysis. Quintile regression models assessed the association of demographic and clinical variables with length of stay.

**Results:** Among 3513 trauma patients, road traffic crashes (RTCs) had a higher percentage of injuries with the Glasgow Coma Scale (GCS) between 9 and 12 (1.7%) compared to falls (0.3%) (*P*<0.001). Falls caused more moderate cases with injury severity scores (ISS) ranging from 9 to 15 (22.7%) than RTCs (17.1%) (*P*<0.001). RTC-related injuries required more ventilation (2.7%) and intensive care unit (ICU) admissions (11.1%) than falls (*P*<0.001). After adjusting for age, GCS, ISS, and body region, fall had a median length of stay nine hours shorter than RTCs (95% CI = -16.2, -1.8).

**Conclusion:** Significant injury pattern differences were observed between RTCs and falls. RTCs had higher frequencies of injuries resulting in GCS scores between 9 and 12, while falls had higher frequencies of moderate ISS scores. In addition, patients with RTC-related injuries required more mechanical ventilation and ICU admissions. Moreover, after adjusting for various factors, patients with RTC-related injuries had a significantly longer hospital stay compared to those with fall-related injuries.

## Background

 Annually, trauma is responsible for causing the deaths of around five million people. These fatalities represent almost 9% of all global deaths.^1^ People under 44 account for about half of injury-related deaths globally.^1^ According to the 2019 World Health Organization (WHO) report, low- and middle-income countries experienced a significant number of fatalities due to road traffic crashes (RTCs) every year.^2^ Iran, a lower-middle-income country with a population of over 87 million people, confronts tremendous challenges in dealing with high rates of mortality and morbidity caused by trauma.^3^ Based on the WHO global status report on road safety, 1 354 840 RTC-related mortalities occurred in Iran last year.^4^ RTCs account for 5.63% (95% UI: 4.83‒6.58%) of all disability-adjusted life years in the Iranian population in 2019.^5^

 The trauma registry serves as a crucial instrument for documenting epidemiology, administration, and trauma outcomes, aiding in elevating the standard of care and preventing future incidents.^6^ The widespread implementation of trauma registries has resulted in declining mortality and morbidity rates in developed countries.^6^ Nevertheless, obtaining precise information about trauma patients in low- and middle-income countries is difficult due to multiple obstacles, including insufficient trauma registries, inadequate infrastructure, and limited trained healthcare personnel.^7,8^

 This study assessed the epidemiology of injuries in trauma patients admitted to one of the main National Trauma Registry of Iran (NTRI) collaborating centers.

## Methods

 This study was conducted utilizing the NTRI’s data from September 17, 2016, to March 1, 2023. The NTRI’s registration process, questionnaire, and data quality evaluation have been discussed elsewhere.^9-12^ All collaborating centers used the same inclusion criteria, which were hospitalization for more than 24 hours, post-trauma death on the first day of admission, or transfer from other hospitals’ intensive care units (ICUs). Any patient admitted to Imam Hossein Hospital, Shahroud, Iran, between 2016 and 2023 and who met the inclusion criteria was included in this study.

 Data collection was divided into several phases. Initially, eligible patients were identified through the hospital information system. Next, three trained interviewers filled out the questionnaire by interviewing the patient or the patient’s companion or using the patient’s hospital profile as the last source for retrieving information. The patient’s informed consent was obtained in compliance with ethical considerations. The collected data were uploaded onto the NTRI’s online portal. Then, expert reviewers carefully double-checked the data for both completeness and accuracy.

 Demographic data, injury characteristics, emergency department information, injury severity indicators, diagnosis, and outcomes were examined in this study. The causes of injury were divided into five main groups, including RTCs, blunt injuries, falls, stabs and/or cuts, and others (i.e., burns, poisoning, gunshots, animal bites, traumatic asphyxia, electrical damage, and blast injury). The abbreviated injury scale (AIS) was applied for grading injury severity. Then, the injury severity scores (ISS), in particular injured body regions, were calculated using AIS. In this study, ISS scores of 1‒8, 9‒15, and 16 > were defined as mild, moderate, and severe injuries, respectively.^13^ In addition, multiple trauma was defined as having at least two injured body regions with AIS > 2.^14^ The Glasgow Coma Scale (GCS) was categorized into three groups, namely, mild (3‒8), moderate (9‒12), and severe (13‒15) head injuries, based on the literature.^13,15^ Low systolic blood pressure was defined as SBP less than 90 mmHg. Tachycardia was identified as a heart rate of 100 beats per minute or higher. Further, tachypnea was determined by a respiratory rate of 20 breaths per minute or more, and a SpO_2_ level below 90% indicated hypoxemia.

###  Statistical analysis

 Nominal and categorical variables were described using numbers and percentages, as well as means and standard deviations (SD). The median and interquartile range (IQR) were used to describe the length of stay. The chi-square test and Fisher’s exact test were utilized to assess the association between nominal and categorical variables. The median length of stay among causes of the injury was compared using the Kruskal-Wallis H test. Univariate and multiple quantile regression models were employed to determine the predictors of length of stay. The significance level used in the tests was set at a *P* value < 0.05. The obtained data were analyzed by the Stata software, version 14.0 (Stata Corp., College Station, TX, USA).

## Results

 A total of 3513 trauma patients, with 2,578 (73.4%) of them being males, were included in this study. Patients’ ages ranged from 1 to 96 years, with a mean ( ± SD) of 35.12 ( ± 20.04) years. In our trauma patient population, there was a significantly higher percentage of male adults (71.6%) compared to female adults (62.7%) (*P* < 0.001). Additionally, the proportion of married females among the trauma patients was significantly higher than that of married males (70.1% versus 56.6%) (*P*< 0.001). Furthermore, significantly higher percentages of blunt trauma (11.1%), stab/cut injuries (13.5%), and falls (22.0%) were found in males compared to females (*P* < 0.001). Further data on the baseline characteristics of trauma patients are delineated in Table 1.

**Table 1:** Baseline characteristics of trauma patients by gender

**Variables**	**Male (n=2578)**		**Female (n=935)**		**Total (n= 3513)**	***P*** ** value**
	**Number**	**Percent**	**Number**	**Percent**		
Age (y)						
Pediatric ( < 18)	566	22.0	169	18.1	735	0.001
Adult (18‒64)	1,845	71.6	586	62.7	2,431	
Geriatric ( ≥ 65)	166	6.4	179	19.2	345	
Missing	1		1		2	
Education						
No formal education	229	8.9	236	25.2	465	0.001
Primary education	512	19.9	251	26.8	763	
Secondary education	1,605	62.3	357	38.2	1,962	
Higher education	231	9.0	91	9.7	322	
Missing	1		0		10	
Marital status						
Single	1,107	43.0	229	24.5	1,336	0.001
Married	1,458	56.6	655	70.1	2,113	
Divorced/widow	11	0.4	50	5.4	61	
Missing	2		1		3	
Hospital transportation						
Ambulance	1,350	52.5	501	53.8	1,851	0.308
Private car	1,208	47.0	430	46.1	1,638	
Others	11	0.4	1	0.1	12	
Missing	9		3		12	
Cause of injury						
Road traffic crashes	1,285	49.8	461	49.3	1,746	0.001
Blunt	285	11.1	55	5.9	340	
Fall	568	22.0	329	35.2	897	
Stab/cut	348	13.5	66	7.1	414	
Others	92	3.6	24	2.6	116	

 ISS ≥ 16 was reported in 26 (0.7%) patients, and 24 (0.7%) patients had GCS scores between 3 and 8. Our analysis showed no statistically significant differences in patients’ four primary vital signs on the day of admission between those with different causes of injuries. There were no significant differences in the proportion of patients with GCS scores of 3 to 8 and ISS scores above 16 across various injury causes. Injuries resulting from RTCs led to a higher number of cases with GCS scores between 9 and 12 (1.7%) in comparison to fall-related injuries (0.3%) (*P*< 0.001). Falls resulted in a greater number of moderate cases, with ISS scores ranging from 9 to 15 (22.7%) compared to cases involving RTC (17.1%), blunt (5.0%), and stab/cut (2.2%) injuries (*P* < 0.001). Additionally, RTCs resulted in more moderate cases than blunt and stab/cut injuries (*P*< 0.001). Further data on the clinical characteristics of different causes of injuries are outlined in Table 2.

**Table 2:** Clinical Characteristics of the Cause of Injuries

**Variables**	**Road Traffic Crashes**		**Blunt**		**Fall**		**Stab/Cut**		**Others**		**Total**	***P*** ** value**
	**No.**	**%**	**No.**	**%**	**No.**	**%**	**No.**	**%**	**No.**	**%**		
Systolic blood pressure (mm Hg)												
> 90	1,685	96.7	326	95.9	858	95.7	396	95.7	107	92.2	3372	0.146
≤ 90	58	3.3	14	4.1	39	4.3	18	4.3	9	7.8	138	
Pulse rate (beat/min)												
< 100	1,496	85.8	299	87.9	773	86.2	372	89.9	95	81.9	3035	0.115
≥ 100	247	14.2	41	12.1	124	13.8	42	10.1	21	18.1	475	
Respiratory rate (/min)												
< 20	1,656	95.0	331	97.4	865	96.4	402	97.1	109	94.0	3363	0.079
≥ 20	87	5.0	9	2.6	32	3.6	12	2.9	7	6.0	147	
O_2_ saturation (%)												
< 90	16	0.9	1	0.3	7	0.8	1	0.2	3	2.6	28	0.103
≥ 90	1,727	99.1	339	99.7	888	99.2	412	99.8	113	97.4	3479	
Glasgow coma scale												
3‒8	18	1.0	1	0.3	3	0.3	0	0.0	2	1.7	24	0.001
9‒12	29	1.7	2	0.6	3	0.3	0	0.0	4	3.4	38	
13‒15	1,693	97.3	337	99.1	890	99.3	414	100.0	110	94.8	3444	
Injury severity score												
Mild (1‒8)	1,429	81.8	322	94.7	689	76.8	404	97.6	108	93.1	2952	0.001
Moderate (9‒15)	298	17.1	17	5.0	204	22.7	9	2.2	7	6.0	535	
Severe ( ≥ 16)	19	1.1	1	0.3	4	0.4	1	0.2	1	0.9	26	

 Extremity injuries and multiple traumas were the most common injuries among all trauma causes. In both RTC- and fall-related injuries, the most commonly injured body regions that resulted in an AIS score of 3 or higher were the extremities, followed by multiple traumas (Table 3).

**Table 3:** Assessment of severe injury (abbreviated injury score ≥ 3) in different body regions

**Body region**	**Road traffic crashes**	**Blunt**	**Fall**	**Stab/Cut**	**Others**	**Total**
Head/neck/face	26	0	6	1	1	34
Thorax	0	0	1	1	1	3
Abdomen	0	0	0	0	1	1
Spine	0	1	0	0	0	1
Extremities	103	10	153	1	0	267
Multiple trauma	177	8	51	7	3	246
Unknown	0	0	0	0	1	1

 The outcomes for various injury causes are provided in Table 4. A total of 296 (8.4%) patients were admitted to the ICU, and 62 (1.8%) needed mechanical ventilation. A total of 24 (0.7%) cases resulted in death due to trauma or trauma-related complications. The mean ( ± SD) age of patients who died due to trauma was 40.71 ( ± 25.57) years, and 75.0% of them were females. Of the patients who died due to trauma, 18 (75.0%) were ICU admitted. There were no significant differences in in-hospital mortality frequencies between different causes of injuries (*P* = 0.13). RTCs had a statistically significantly higher frequency of ICU admission (11.1%) in comparison with fall (7.4%), stab/cut (4.1%), and blunt (3.5%) injuries (*P* < 0.001). Moreover, patients with RTC-related injuries required mechanical ventilation (2.7%) more often than those with fall (1.0%) and stab/cut (0.2%) injuries (*P*< 0.001). The median (IQR) length of stay in the patients was 86.0 (87.7) hours. Patients with injuries related to RTCs had a longer hospital stay of 92.0 (94.0) hours compared to those with stab/cut (median: 65.0, IQR: 49.5) and blunt (median: 66.0, IQR: 64.0) injuries (*P* < 0.01). Patients with falls had a median length of stay (IQR) of 87.0 (92.0) hours, whereas stab/cut patients had a median length of stay (IQR) of 65.0 (49.5) hours (*P*< 0.01).

**Table 4:** The association between injury outcomes and the cause of injuries

**Injury outcome**	**Road traffic crashes**	**Blunt**	**Fall**	**Stab/Cut**	**Others**	**Total**	***P *** **value**
ICU admission	193	12	66	17	8	296	0.001
Mechanical ventilation	47	2	9	1	3	62	0.001
In-hospital mortality	16	1	5	0	2	24	0.132
Median length of stay hours (IQR)	92.0	66.0	87.0	65.0	83.0	86.0	0.001

*Note*. ICU: Intensive care unit; IQR: Interquartile range.

 The results of the multiple quintile regression model indicated statistically significant associations between age, ISS, GCS, cause of injury, body region, and length of stay. After adjusting for age, GCS, ISS, and body region, the median length of stay in patients with fall-related injuries was nine hours less than in patients with RTC-related injuries (95% CI: -16.2 ‒ -1.8; Table 5).

**Table 5:** Quintile regression of the relationship between length of stay (hours) and characteristics of trauma patients

**Variables**	**Crude β (95% CI)**	**Adjusted β (95% CI)**
Age (y)		
< 18	Ref.	Ref.
18‒64	15 (8.2, 21.7)	12 (5.0, 18.9)
≥ 65	47 (36.6, 57.4)	30 (18.7, 41.3)
Injury severity score		
1‒8	Ref.	Ref.
9‒15	81 (72.8, 89.2)	65 (56.7, 73.3)
≥ 16	229 (194.8, 263.2)	199 (165.9, 232.1)
Glasgow coma scale		
13‒15	Ref.	Ref.
9‒12	220 (193.5, 246.5)	168 (140.5, 195.5)
3‒8	249 (215.8, 282.2)	186 (151.7, 220.3)
Cause of injury		
Road traffic crashes	Ref.	Ref.
Blunt	-26 (-34.3, -17.7)	-21 (-30.9, -11.1)
Fall	-5 (-10.8, 0.8)	-9 (-16.2, -1.8)
Stab/cut	-27 (-34.7, -19.3)	-23 (-32.2, -13.8)
Others	-9 (-22.4, 4.4)	-5 (-25.3, 15.3)
Body region		
Extremities	Ref.	Ref.
Head/neck/face	7 (-1.1, 15.1)	2 (-8.2, 12.2)
Thorax	18 (0.2, 35.8)	11 (-11.3, 33.3)
Abdomen	14 (-9.1, 37.1)	19 (-9.7, 47.7)
Spine	47 (30.1, 63.9)	48 (27.1, 68.9)
Multiple trauma	15 (10.1, 19.9)	12 (5.8, 18.2)
Unknown	4 (-16.1, 24.1)	4 (-27.1, 35.1)

*Note*. CI: Confidence interval.

## Discussion

 The study findings revealed that RTCs were the leading cause of trauma, followed by falls, which aligns with the findings of other studies conducted in similar settings. A study on nearly three million injury cases in Iran identified RTCs and falls as the primary causes of injury.^16^ Another study conducted in Egypt over eight years reported that falls and transport accidents were the most prevalent types of injuries ^17^. According to the WHO’s report on injuries and violence, road traffic injuries and falls were among the top leading causes of deaths related to injuries.^18^

 Patients involved in RTCs had a lower mean age compared to those who fell in our study. This finding conforms to the results of previous studies.^12,19,20^ A survey of geriatric trauma cases showed that individuals who experienced falls had a greater average age when compared to those who sustained injuries from other mechanisms of injury.^21^ In a study performed in Georgia, falls were the primary cause of hospitalization among elderly trauma patients.^22^

 Our findings revealed significant associations between the cause of trauma and GCS, ISS, and AIS ≥ 3. The ISS scores were higher for falls and RTCs compared to blunt and stab/cut injuries. This result is consistent with the findings of a study by Saeednejad et al, which examined 1,817 registered trauma patients admitted to Sina Hospital between 2016 and 2018.^12^ According to Sterling et al, falls were a frequent cause of severe injury among the elderly.^20^ Unlike other causes, falls resulted in more injuries with an AIS ≥ 3 in the extremities. RTCs accounted for the greatest proportion of injuries with an AIS of 3 or higher in the head/face/neck and multiple traumas in our study. A study conducted on geriatric fall-related trauma patients in Canada demonstrated that older individuals were more likely to sustain injuries to the head, face, and lower limbs.^23^ Ameri et al found significant variations in age, gender, GCS, ISS, and AIS in both upper and lower limbs and multiple injuries from different causes of injuries. In addition, patients who suffered from road accidents and falls had a significantly higher mean ISS and AIS in extremities than those who experienced other forms of trauma.^24^

 The findings of our study indicated that patients who experienced RTCs were more frequently admitted to the ICU and required ventilation compared to those with other types of injuries, which corroborates the results of Ameri et al.^24^

 Based on our findings, length of stay was influenced by independent factors such as age, ISS, GCS, cause of injury, and body regions. A survey performed on trauma patients in Southwestern Iran’s trauma center delineated some factors, including gender, age, site of injury, infections, and surgery, which may impact the length of hospital stay in patients.^25^ In the study of Andersen et al, patients’ length of stay was prolonged due to a combination of factors, including the severity of the injury.^26^

 This study had some limitations. It was conducted in a single center; therefore, it may not be representative of the entire population of Iran. Furthermore, the limited number of injuries (including zero cases in certain body regions) with AIS scores of 3 or higher across various trauma causes posed a challenge in calculating a *P* value and determining the statistical significance of the observed difference.

**Highlights**

- RTCs were the leading cause of injury, followed by falls.
- Injury patterns differed between RTCs and falls, including severity scores (GCS and ISS) and affected body regions.
- RTCs had more cases with GCS scores of 9‒12 and head/face/neck injuries with AIS ≥ 3.
- Falls had more extremity injuries with AIS ≥ 3 and a higher frequency of moderate ISS scores.
- Patients with RTC-related injuries had more mechanical ventilation, higher ICU admissions, and longer hospital stays compared to those with falls.

## Conclusion

 Our results confirmed that most trauma cases were observed in men, and RTCs were the leading cause of injury, followed by falls. Patients with fall-related injuries were generally older compared to those with RTCs. There were significant differences in injury patterns between RTCs and falls, including a higher percentage of injuries resulting in GCS scores between 9 and 12 for head/face/neck injuries with AIS ≥ 3 in RTCs, more extremity injuries with AIS ≥ 3, and a higher frequency of moderate ISS scores in falls. Patients with RTC-related injuries also had a higher frequency of mechanical ventilation and ICU admissions. Furthermore, after adjusting for age, ISS, GCS, and body region, patients with fall-related injuries had a significantly shorter length of stay than those with RTC-related injuries.

## Acknowledgments

 We are grateful to all the colleagues who participated in this study.

## Authors’ Contribution

**Conceptualization:** Payman Salamati, Mahgol Sadat Hassan Zadeh Tabatabaei.

**Data curation:** Salman Daliri, Khatereh Naghdi.

**Formal analysis:** Vali Baigi.

**Funding acquisition:** Mohammadreza Zafarghandi.

**Investigation:** Armin Khavandegar, Salman Daliri.

**Methodology:** Vali Baigi.

**Project administration:** Payman Salamati, Mohammadreza Zafarghandi.

**Resources:** Mohammadreza Zafarghandi.

**Software:** Khatereh Naghdi.

**Supervision:** Vafa Rahimi-Movaghar, Payman Salamati.

**Validation:** Vafa Rahimi-Movaghar.

**Visualization:** Mahgol Sadat Hassan Zadeh Tabatabaei, Sara Mirzamohamadi.

**Writing–original draft:** Mahgol Sadat Hassan Zadeh Tabatabaei.

**Writing–review & editing:** Armin Khavandegar.

## Competing Interests

 The authors declare that they have no potential conflict of interests related to this study.

## Ethical Approval

 The Ethics Committee of Sina Hospital, Tehran University of Medical Sciences, has approved this project with the approval number REC.1399.090, dated January 3, 2021.

## Funding

 The Sina Trauma and Surgery Research Center financially supported the study under the project of the NTRI.
